# Burnout syndrome in Primary Health Care

**DOI:** 10.47626/1679-4435-2021-938

**Published:** 2024-02-16

**Authors:** Érika Maria Izaias, Rita de Cássia de Marchi Barcellos Dalri, Maria Lúcia do Carmo Cruz Robazzi, Fábio de Souza Terra, Elaine de Jesus Gusmão Sampaio

**Affiliations:** 1 Programa de Mestrado Profissional de Tecnologia e Inovação em Enfermagem, Escola de Enfermagem de Ribeirão Preto, Universidade de São Paulo (USP), Ribeirão Preto, SP, Brazil; 2 Escola de Enfermagem de Ribeirão Preto, USP, Ribeirão Preto, SP, Brazil; 3 Escola de Enfermagem, Universidade Federal de Altenas, Altenas, MG, Brazil; 4 Enfermagem, Universidade Paulista, Araraguara, SP, Brazil

**Keywords:** burnout, professional, occupational health, primary health care, esgotamento profissional, saúde do trabalhador, atenção primária à saúde

## Abstract

**Introduction:**

The labor situation is considered essential for social follow-up; however, the contradiction between its importance and the psychic changes it can cause to workers should be considered.

**Objectives:**

To check the presence of burnout syndrome in Primary Health Care workers in a municipality in the interior of the state of São Paulo and to check the association with the sociodemographic and labor data of these workers.

**Methods:**

Exploratory cross-sectional and quantitative study with 74 workers and developed from July to October 2020. Data were collected using an instrument for sociodemographic and labor characterization and for measurement of the syndrome, the Oldenburg Burnout Inventory already validated in Brazil. Descriptive statistics, frequency, percentage, measures of central tendency and dispersion were used; Fisher's exact test was applied to check the association between the studied variables and the burnout syndrome, considering the significance level of p < 0.05. The obtained data were discussed using the existing literature.

**Results:**

Among the participants, 20.3% had no changes, 18.9% showed distance, 16.2% had exhaustion, and 44.6% had burnout syndrome. There was statistical significance for the variable unit of work (p < 0.014).

**Conclusions:**

The importance of studying labor mental health in Primary Health Care is because of the constant exposure of workers to psychosocial risk situations in social settings and their instabilities. Knowledge of the conditions allows for intervention actions in the environments and makes it possible to face the problems.

## Abstract (pt)

**Introdução:**

A conjuntura laboral é considerada indispensável ao seguimento social, entretanto, há que se considerar a incongruência entre sua importância e as alterações psíquicas que pode ocasionar aos trabalhadores.

**Objetivos:**

Verificar a presença da síndrome de *burnout* em trabalhadores da Atenção Primária à Saúde de um município do interior do estado de São Paulo e verificar sua associação com os dados sociodemográficos e laborais desses trabalhadores.

**Métodos:**

Estudo exploratório, transversal e quantitativo, direcionado a 74 trabalhadores e desenvolvido de julho a outubro de 2020. Para a coleta de dados, utilizou-se instrumento para caracterização sociodemográfica e laboral e, para mensuração da síndrome, o Oldenburg Burnout Inventory, já validado no Brasil. Foram realizadas estatísticas descritivas, frequência, percentual, medidas de tendência central e dispersão; utilizou-se o teste exato de Fisher para verificar a associação entre as variáveis estudadas e a síndrome de *burnout,* considerando o nível de significância p < 0,05. Os dados obtidos foram discutidos mediante a literatura existente.

**Resultados:**

Dentre os participantes, 20,3% não apresentaram alterações, 18,9% apresentaram distanciamento, 16,2% apresentaram exaustão e 44,6%, síndrome de *burnout.* Houve significância estatística para a variável unidade de trabalho (p < 0,014).

**Conclusões:**

Estudar a saúde mental laboral na Atenção Primária à Saúde tem importância, uma vez que há constante exposição a situações de riscos psicossociais nos cenários sociais, que apresentam instabilidades. O conhecimento das condições possibilita ações de intervenção nos ambientes e viabiliza o enfrentamento dos problemas.

## INTRODUCTION

Work has objective and subjective dimensions; it is capable of both fulfilling social needs and mediating the relationship between humankind and nature. It is considered indispensable to social life, as professional relationships help to minimize uncertainty and dissatisfaction, as they strengthen personal aspects that permeate the field of achievement instilled in every human being.^1^

Considering that work is an intrinsic practice of individuals, its relationship with the health-disease process should also be considered, as it is both a maintainer and a precarious factor that exposes human beings to adverse health situations.^2^

Health care industry is a wide-ranging labor scenario, with possibilities for developing work processes in both the public and private sectors. The public sector has Primary Health Care (PHC) as its gateway, which advocates comprehensive care to prevent, treat, and promote community health.^3,4^

Technical knowledge and specific skills are mandatory to PHC personnel. However, only scientific knowledge is not enough to fulfill the attitudes expected by PHC personnel. Individuals have countless skills and individualities in their intellect, so PHC personnel ought to practice respect for different patients in different settings, while not trying to nullify the community in pre-existing analyses.^3^

However, to meet the requirements of this job, health personnel can be negatively affected by workload and overload, pressure at work and due to institutional organization, which can lead to psychosocial risks and their repercussions, as well as other possible situations that affect mental health.^4^

Burnout syndrome (BS) stands out among the mental health disorders affecting healthcare personnel, as a response to prolonged exposure to stressful situations that lead to suffering, emotional exhaustion, depersonalization, and feelings of low personal accomplishment. Workplace management models and other conditions can lead to psychosocial risks and unfavorable impacts on their mental health.^4^

In this context, this study aimed to identify BS in PHC personnel in a municipality in inland São Paulo, Brazil, and determine its association with the participants' sociodemographic and occupational data.

## METHODS

This is an exploratory, descriptive, cross-sectional study with a quantitative approach, conducted in a municipality located in inland São Paulo. The population consisted of 96 PHC personnel; after applying the selection criteria (having worked in PHC for at least 6 months and being present on the dates scheduled for data collection), a sample of 74 workers was obtained.

This study followed the ethical guidelines for research involving human beings in accordance with Resolution No. 466 of December 12, 2012 of the Conselho Nacional de Saúde (CNS - National Health Council)^5^ and was approved by the Research Ethics Committee with opinion number 4.078.163.

The sociodemographic characteristics questionnaire has 19 questions. To measure BS, we used the Oldenburg Burnout Inventory (OLBI), developed by Demerouti in 1999 and validated in Brazil in 2013 by Schuster & Dias,^6^ a 13-item tool that assesses exhaustion and work detachment. The answers are graded from 1 to 4, where 1 is "strongly disagree" and 4 is "strongly agree."^6^

The collected data were double-entered. Descriptive statistics, frequency, and percentage for the qualitative variables, and measures of central tendency (mean and median), and dispersion (SD, standard deviation) for the numerical variables were then calculated. The association between sociodemographic and work-related variables and BS was analyzed with Fisher's exact test, with a significance level of p < 0.05.

## RESULTS

A total of 74 healthcare personnel participated in the study, with a mean age of 38 years, mostly women (87.8%), with a partner (48.6%), 1 to 2 children (54.1%), college degree (40.5%), civil service contract (83.8%), and an overall working time between 6 and 10 years at the healthcare unit. There was a low percentage of overtime (5.5%) and, during data collection, 86.5% of participants reported working up to 40 hours a week.

The group with most workers was community health workers (16.2%), followed by vector agents (9.5%), nursing technicians and aides (9.5%) and physicians (9.5%). Cleaning workers made up 8.1%, and 6 clerical workers (8.1%). Nurses numbered 4.1%, dentists (4.1%), dental assistants (4.1%), and managers (4.1%). Speech therapists were 2.7% of the sample, together with physical therapists (2.7%), interns (2.7%), pharmacists (2.7%), administrative assistants (2.7%), and social workers (2.7%). The following professions had 1 participant: psychologist (1.4%); nutritionist (1.4%); permanent education (1.4%); pharmacy technician (1.4%); and driver (1.4%).

Table 1 shows the description of the workers' responses to the questions that led to the results of detachment and exhaustion.

**Table 1:** Distribution of responses from Primary Health Care personnel according to the Oldenburg Burnout Inventory (OLBI), in a municipality in São Paulo, Brazil, 2020 (n = 74)

Questions	Completely disagree (1)		Disagree (2)		Agree (3)		Completely agree (4)	
	n	%	n	%	n	%	n	%
Detachment								
1. I often do new and interesting things in my work.	2	2.7	37	50.0	34	45.9	1	1.4
2. I talk more and more negatively about my work.	10	13.5	55	74.3	7	9.5	2	2.7
3. I have been doing my job almost mechanically lately.	2	2.7	36	48.6	33	44.6	3	4.1
4. I see my work as a positive challenge.	11	14.9	59	79.7	3	4.1	1	1.4
5. Over time I have become disinterested in my work.	15	20.3	37	50.0	21	28.4	1	1.4
6. I feel increasingly committed to my work.	8	10.8	39	52.7	27	36.5	0	0.0
7. I often feel fed up with my tasks.	1	1.4	28	37.8	37	50.0	8	10.8
Exhaustion								
8. Some days I feel tired even before I get to work.	3	4.1	27	36.5	33	44.6	11	14.9
9. After work I need more time to feel better than I used to.	3	4.1	39	52.7	22	29.7	10	13.5
10. I can cope very well with the pressures of my job.	5	6.8	52	70.3	14	18.9	3	4.1
11. During my work I feel emotionally drained.	2	2.7	40	54.1	30	40.5	2	2.7
12. After work I have energy for leisure activities.	4	5.4	45	60.8	21	28.4	4	5.4
13. After work I feel tired and drained.	1	1.4	35	47.3	36	48.6	2	2.7

Source: Prepared by the author (2021), based on the OLBI scale by Schuster & Dias.^6^

For statistical analysis, the Fisher's exact test was used to verify associations between the variables, considering a significance level of p < 0.05. The variable workplace differed from the others as it was statistically significant (p = 0.014).

Among women, 16.9% had no BS, while among men 44.4% had no BS. Detachment was found in 18.5% of women and 22.2% of men. Exhaustion was not found in men, but 18.5% of women did. As for BS, 46.2% of women and 33.3% of men had it.

Among 15 workers aged between 19 and 29, 46.7% had BS. The group aged 30 to 39 totaled 31 workers, 51.6% of whom were classified as having BS. The group aged 40 to 49 had 14 workers and 35.7% had BS. The population aged 50 and over had a total of 14 participants, and 35.7% had BS.

Of the 24 participants who reported having no children, 50% had BS. Of the 40 workers who reported having 1 to 2 children, 45% had BS. The population that reported having 3 or more children included 10 people, and 30% had BS.

Among the 6 participants with elementary school, 50% had BS. Among the 22 workers who had completed high school, 54.5% had BS; 30 respondents had completed an undergraduate degree, 30% had BS. In addition, 16 participants had reported having a postgraduate level of education, and 56.3% had BS.

As for the weekly workload, 64 workers reported working between 20 and 40 hours a week, and 43.8% scored for BS. Another 10 participants reported working more than 40 hours a week and the associations showed 50% for BS.

Of the 10 workers who had been on vacation for the last 30 days prior to data collection and resumed work, 3 had BS scores. Of the 64 participants who had not been on vacation in the 30 days prior to data collection, BS scores were higher than those who had been on vacation: 30 (46.9%) had BS.

Participants were asked how long they had worked at the healthcare unit and 30 reported having worked from 6 months to 5 years, and 14 (46.7%) had a BS score.

A total of 26 participants reported having worked between 6 and 10 years, and 13 (50%) had a BS score. Another 10 participants said they had worked between 11 and 20 years at the institution, and 1 (10%) had a BS score.

Of the 6 participants who reported having worked for 21-29 years, 3 (50%) had a BS score. Both respondents who reported more than 30 years in the job also scored for BS.

As for the length of time they had worked in PHC, 35 respondents reported 6 months to 5 years, and 18 (51.4%) had a BS score.

A total of 32 workers had worked in PHC for between 6 and 15 years and 10 (31.3%) scored for BS.

Of the 6 workers who had worked in PHC for 16 to 20 years, 4 (66.7%) had BS. Only 1 worker had worked in PHC for more than 20 years and this also had BS.

As for their time working in the health care unit, 51 workers reported working from 6 months to 5 years; 18 from 6 to 10 years; and 5 for more than 10 years. The analyses of these 3 groups showed a higher percentage of BS compared to the other categories, indicating a tendency for BS episodes to occur in all the time periods assessed. These showed BS scores of 45.1%, 44.4%, and 40%, respectively.

When asked about how many jobs they had, participants responded 1, 2 or more than 2. The first option was answered most frequently and resulted in 60 responses, and 26 (43.3%) had a BS score.

The remaining 14 participants reported having 2 or more jobs and the findings showed that 7 (50%) had BS.

As for their employment relationship, 62 workers were civil servants, and 30 (48.4%) had a BS score.

A selection process was reported by 8 participants, and 2 (25%) were found to be compatible with BS.

A total of 3 workers reported a contracted position, of which 1 (33.3%) had a BS score.

The variable workplace was divided into 5 categories, the first 3 had the same profile of care and were congruent in terms of the composition of the teams, as they were set up as Family Health Strategy units, and therefore had a similar number of workers.

Of the 3 units with the same profile of care, Unit 1 had a total of 12 responses and only 2 workers (16.7%) were found to have a BS score.

Unit 2 had the highest percentage of mental health problems; 15 workers worked in this unit and 11 (73.3%) had a BS score.

Unit 3 had a total of 11 responses, and 5 (45.5%) had a BS score.

Unit 4 was a Basic Health Unit and Specialty Unit; it included vector prevention and health surveillance services and, therefore, a different type of care from the first 3 units. This unit had 31 participants, and 11 (35.5%) had BS.

Finally, Unit 5 had 5 workers who provided interdisciplinary care and supported the health units to broaden the care service provided; 80% in this group had a BS score.

Monthly family income ranged from 1 to 11 or more Brazilian minimum wages, considering the amount of R$ 1,045.00 per month at the time of data collection, and it was subdivided into 4 categories according to the answers found: 40 people reported a monthly family income of 1 to 3 minimum wages, 40% had a BS score; 25 participants reported a monthly family income of 4 to 6 minimum wages, 52% had a BS score. A total of 4 workers reported a monthly family income of 11 or more minimum wages, and 1 (25%) had a BS score.

The occupational groups were divided into 4 categories; the largest was the care services group, with 53 workers, and 24 (45.3%) had a BS score.

Clerical services group had 11 participants, and 6 (54.5%) had a BS score.

The support services group had 4 respondents, and none had a BS score.

Finally, of 6 general service workers, 3 (50%) had a BS score.

As for the associations, the variable workplace was statistically significant (p = 0.014), with no statistical significance in the other associations. In general, among the 74 workers included in the OLBI survey, 20.3% had no BS, 18.9% had detachment, 16.2% had exhaustion, and 44.6% had BS.

## DISCUSSION

The predominance of women in healthcare jobs is known as the feminization of work and was also seen in this study, accounting for 87.8% of women. This makes us reflect on the strengthening of this group in the workforce and the move towards new personal and professional expectations, even when faced with the challenges of an extended working day.^7^

The highest percentage of individuals who scored for BS was between 30 and 39 years old (41.9%), which may be related to greater vulnerability to stressful situations due to less maturity and less control of emotions.^8^ On the other hand, the literature also suggests that the older age group may find it less easy to adapt to the workplace and, in this case, trigger changes in the mental health of individuals;^9^ both theories point to the importance of considering age in studies on the subject.

Childbearing and, consequently, family relationships can be seen as protective factors and are related to lower mental health problems in workers.^10^ However, in this study, among the participants who reported having 1 to 2 children, 54.1% of them had a BS score, showing that the highest percentage was observed in those who had children, rather than those who did not.

As for educational background, a study conducted in an emergency room of a university hospital in Paraná, Brazil, showed that people with a higher level of education were more likely to develop BS;^11^ similar to what was found in this study, in which workers with a university degree were more likely to be affected by BS.

As for professional experience, most workers reported a mean of 6 to 10 years working in their institution and the same mean was found in PHC. A Brazilian study conducted in Goiás reported that working time was directly related to episodes of anxiety.^12^ In another study conducted in Minas Gerais, Brazil, workers who were younger and had been working longer had a higher level of stress.^13^

The discrepancy in these findings may reinforce the concept that the subjectivity of each worker should be analyzed individually and/or in terms of the specificities of the team they are involved with, since numerous contributing factors to illness at work can be hidden behind positive stigmas in relation to stability and professional experience.^14^ This study found that BS was present in all age groups, which corroborates the findings in the literature.

This study was conducted in a public sector facility; it is worth noting that most workers interviewed (83.8%) reported having been recruited through a civil servant examination. The literature validates findings that a lack of job stability facilitates the occurrence of changes in thoughts and feelings, which can affect the worker's psychological situation.^15^

However, it is worth recalling that civil servants often report living with a sense of demerit related to their working condition, as they are often seen as favored due to their stability.^16^ As such, the possibility of becoming ill at work is underestimated, a fact that alerts researchers because, even with a stable contract, they are still immersed in a work environment that is subject to instability in the working process and the subjectivity of each worker.^17,18^

This study found that most workers (54.1%) had a monthly family income between 1 and 3 Brazilian minimum wages and considerable tendency to BS. However, this tendency was also seen in groups with higher income, which may be connected to a finding that, when assessing income and job, a large part of life is consumed in the workplace and, sometimes, the pay and the workplace do not match the extent of the services provided, which can be a precursor to emotional distress due to worker dissatisfaction and a feeling of low recognition.^19^

The results presented in this study showed that among the participants, healthcare personnel stood out (71.6%) and, notable in this category is the existence of multi-professional characteristics. Given the above, the discussion turns to the importance of interdisciplinarity in the services provided within PHC and interdisciplinary practice makes it possible to provide comprehensive care in line with the population's health needs.^20^

Workers affected by BS manifest characteristics peculiar to the process of exhaustion and detachment from work, expressions which are the dimensions of the syndrome addressed by the questionnaire used in this study. Exhaustion involves psychological and physical wear and tear, which may or may not be linked to interpersonal relationships at work. Detachment is seen in the individual's attitudes towards their peers, accompanied by a feeling of low accomplishment at work and low identification with the activity performed.^21^

The variable workplace was statistically significant in this study. Workers' insertion in the institutional environment subjects them to situations that stimulate or weaken their productivity, which is due to variations in this workplace and its interference in the individual's life. Managers should pay attention to the mental health of their staff, as higher levels of exhaustion will also lead to lower levels of work commitment. Therefore, strategies to prevent the mental state of individuals also involve organizational strategies that encourage a cooperative environment and value workers.^22^

Interprofessional relationships develop in the organizational context; differences and similarities between peers result in constant movements of harmonic and/or conflicting situations within the team. When they are confrontational, they can lead to individual behavior and affect the structure of the whole team and, in the case of health services, destabilize the process of providing care. In this sense, a study conducted in southern Brazil with healthcare personnel at a secondary hospital level aimed to propose a job rotation strategy, a systematized rotation with a pre-defined timeframe, to improve the workplace. The result was positive for interpersonal relations, resolving conflicts, and technical improvement. A harmonious team tends to improve work performance and reduce mental health problems.^23^

However, no similar research was found in the PHC setting, which may be related to the model of care that emphasizes the importance of links between teams and the community in order to strengthen longitudinal care.^24^ Given the results found in this study, would this PHC model be beneficial or detrimental to workers' mental health in the long run? While, on one hand, this link provides better team and community communication, and also facilitates care; on the other hand, workers' mental health can be adversely affected by this closeness, as they feel powerless in the long search for improvements in a given group and/or family often unsuccessfully according to local characteristics.^25^

In PHC, there is a strong proximity to numerous situations of social vulnerability and contexts of the community scenario that denote inequalities and weaknesses in the process of health in the face of disease. Faced with these events, workers are more likely to experience psychological changes associated with society's imbalance in relation to behavioral, cultural, and economic conditions.^26^

Tasks management and redistribution strategies, encouraging moments spent sitting and listening, accessibility to management and establishing collective agreements between team members so that the work environment functions in a collaborative way can be beneficial so that PHC workers are not overburdened by the dense community demand arising from care and can perform their duties with greater engagement and enjoyment.^27,28^

Although this study does not aim to assess the impacts of the COVID-19 pandemic, it is important to point out that in 2020, when data was collected, mental health research had increased visibility due to the pandemic, which required management strategies in all social aspects and had a direct impact on health services, which meant that the entire work process had to be readapted. The continuous exposure of physical integrity to the risk factors linked to the new virus has also led to a greater chance of alterations in the mental state of those involved.^29^

Considering the weaknesses caused by damage to workers' mental health and the impact this has on care, we realize that studies on these issues should guide the diagnostic process in institutions. Studies in this respect can act as an indicator of the workplace quality and assess disorders such as BS, and may also become a reference when planning work process facilitators.^30^

This study is limited due to possible influence from the global pandemic scenario and the fight against COVID-19, experienced during data collection; the losses related to sick leave and a smaller overall sample, preventing generalizations on the subject studied.

## CONCLUSIONS

In this study, 45% of workers were affected by mental health problems and there were also significant figures for exhaustion and detachment, totaling the majority of participants in the stages of illness, which shows the need for intervention in the workplace to identify and tackle the problems.

Coping with risk factors and mental health problems can be based on taking breaks from work between tasks, taking advantage of leisure time, seeking support in the family context, finding support in matters related to beliefs, and maintaining clear communication with peers and managers.

It is important to study the mental health scenario of health workers, since they are constantly exposed to psychological risk situations when they provide care to families in the face of numerous social scenarios and their instabilities.

## References

[1] Nobre DFR, Rabiais ICM, Ribeiro PCPSV, Seabra PRC (2019). Burnout assessment in nurses from a general emergency
service. Rev Bras Enferm.

[2] Cardoso AC, Morgado L (2019). Trabalho e saúde do trabalhador no contexto atual: ensinamentos da
Enquete Europeia sobre Condições de Trabalho. Saude Soc.

[3] Junqueira SR (2019). Competências profissionais na estratégia Saúde da
Família e o trabalho em equipe.

[4] Soratto J, Pires DEP, Scherer MDA, Witt RR, Ceretta LB, Farias JM (2020). Family health strategy professional satisfaction in Brazil: a qualitative
study. Texto Contexto Enferm.

[5] Brasil, Ministério da Saúde, Conselho Nacional de Saúde (2012). Resolução nº 466, de 12 de dezembro de 2012.

[6] Schuster M, Dias VV (2018). Oldenburg Burnout Inventory - validação de uma nova forma de
mensurar burnout no Brasil. Cienc Saude Colet.

[7] Salvagni J, Veronese MV, Guerin M, Marques SF (2020). Atividades consideradas "masculinas": mulheres cis e transexuais em busca
de autonomia através do trabalho. Rev Psicol Polit.

[8] Silva CPS, Nunes MAP, Santana VR, Reis FP, Machado Neto J, Lima SO (2015). A síndrome de burnout em profissionais da Rede de
Atenção Primária à Saúde de Aracaju,
Brasil. Cienc Saude Colet.

[9] França FM, Ferrari R (2012). Síndrome de burnout e os aspectos sócio-demográficos
em profissionais de enfermagem. Acta Paul Enferm.

[10] Santos GBM, Lima RCD, Barbosa JPM, Silva MC, Andrade MAC (2020). Cuidado desi: trabalhadoras da saúde em tempos de pandemia pela
Covid-19. Trab Educ Saude.

[11] Jodas DA, Haddad MCL (2009). Burnout Syndrome among nursing staff from an emergency department of a
university hospital. Acta Paul Enferm.

[12] Moura A, Lunardi R, Volpato R, Nascimento V, Bassos T, Lemes A (2018). Fatores associados à ansiedade entre profissionais da
atenção básica. Rev Port Enferm Saude Mental.

[13] Bertussi VC, Junqueira MABB, Giuliani CD, Calçado RM, Miranda FJS, Santos MA (2018). Substâncias psicoativas e saúde mental em profissionais de
enfermagem da Estratégia Saúde da Família. Rev Eletr Enferm.

[14] Huang L, Lin G, Tang L, Yu L, Zhou Z (2020). Special attention to nurses' protection during the COVID-19
epidemic. Crit Care.

[15] Dal'Bosco EB, Floriano LSM, Skupien SV, Arcaro G, Martins AR, Anselmo ACC (2020). A saúde mental da enfermagem no enfrentamento da COVID-19 em urn
hospital universitário regional. Rev Bras Enferm.

[16] Carneiro SAM (2006). Saúde do trabalhador público: questão para a
gestão de pessoas a experiência na Prefeitura de São
Paulo. Rev Serv Publico.

[17] Lancman S, Sznelwar LI (2011). Christophe Dejours: da psicopatologia à psicodinâmica do
trabalho.

[18] Faria RMO, Leite ICG, Silva GA (2017). O sentido da relação trabalho e saúde para os
assistentes em administração de uma universidade pública federal no
Estado de Minas Gerais. Physis.

[20] Mc Hugh MD, Kutney-Lee A, Cimiotti JP, Sloane DM, Aiken LH (2011). Nurses widespread job dissatisfaction, burnout and frustration with health
benefits signal problems for patient care. Health Aff.

[20] Pinho LB, Kantotski LP, Saeki T, Duarte MLC, Sousa J (2007). A integralidade no cuidado em saúde: um resgate de parte da
produção científica da área. Rev Eletr Enferm.

[21] Bernd DC, Beuren IM (2017). A síndrome de burnout está associada ao trabalho dos
auditores internos?. Gest Reg.

[22] Gonçales CA, Gonçales RA (2017). Síndrome de burnout: causas e consequências em diversos
profissionais. Rev Bras Psicol.

[23] Pinhatti EDG, Vannuchi MTO, Sardinha DSS, Haddad MCL (2017). Job rotation of nursing professionals among the sectors of a hospital: a
management tool in conflict resolution. Texto Contexto Enferm.

[24] Pereira RMP, Amorim FF, Gondim MFN (2020). A percepção e a prática dos profissionais da
Atenção Primária à Saúde sobre a Saúde
Mental. Interface.

[25] Dalmolin GL, Lanes TC, Magnago ACS, Setti C, Bresolin JZ, Speroni KS (2020). Prazer e sofrimento em trabalhadores da atenção
primária à saúde do Brasil. Rev Cuid.

[26] Organização Pan-Americana da Saúde (OPAS), Organização Mundial da Saúde (OMS) (2018). Ampliação do papel dos enfermeiros na atenção
primária à saúde.

[27] Porciuncula AM, Venâncio SA, Silva CMFP (2020). Síndrome de burnout em gerentes da Estratégia de Saúde
da Família. Cienc Saude Colet.

[28] Silva JF, Silveira MC, Santos AA, Resende MA, Assis BCS (2020). Síndrome de burnout em profissionais de enfermagem no contexto da
Atenção Básica. Rev Eletr Acervo Saude.

[29] Elhadi M, Msherghi A, Elgzairi M, Alhashimi A, Bouhuwaish A, Biala M (2020). Burnout syndrome among hospital healthcare workers during the COVID-19
pandemic and Civil War: a cross-sectional study. Front Psychiatry.

[30] Hartog F (2020). A Covid-19 e as perturbações no presentismo. ArtCultura.

